# Comprehensive agrobiological assessment and analysis of genetic relationships of promising walnut varieties of the Nikitsky Botanical Gardens

**DOI:** 10.18699/VJGB-23-55

**Published:** 2023-09

**Authors:** Yu.V. Plugatar, I.I. Suprun, S.Yu. Khokhlov, I.V. Stepanov, E.A. Al-Nakib

**Affiliations:** The Order of the Red Banner of Labour Nikitsky Botanical Gardens – National Scientific Center of the Russian Academy of Sciences, Yalta, Republic of Crimea, Russia; North Caucasian Federal Scientific Center of Horticulture, Viticulture, Wine-making, the Functional Scientific Center of “Breeding and Nursery”, Krasnodar, Russia; The Order of the Red Banner of Labour Nikitsky Botanical Gardens – National Scientific Center of the Russian Academy of Sciences, Yalta, Republic of Crimea, Russia; North Caucasian Federal Scientific Center of Horticulture, Viticulture, Wine-making, the Functional Scientific Center of “Breeding and Nursery”, Krasnodar, Russia; North Caucasian Federal Scientific Center of Horticulture, Viticulture, Wine-making, the Functional Scientific Center of “Breeding and Nursery”, Krasnodar, Russia

**Keywords:** walnut, SSR markers, perspective cultivars, collection, genetic diversity, phenotypic evaluation, орех грецкий, SSR-маркеры, перспективные сорта, коллекция, генетическое разнообразие, фенотипическая оценка

## Abstract

Walnut is an important horticultural crop, the production of which ranks second among all nut crops. Despite the significant demand in the domestic market in Russia, the industrial production of walnut fruits in Russia is currently underdeveloped. At the same time, there is a need to update the assortment with new highly productive varieties adapted to local agro-climatic conditions and having high quality nuts that are competitive at the world level. An important issue for the successful implementation of breeding programs is a comprehensive study of the gene pool. In this regard, within the framework of the study, the task was to evaluate promising varieties from the collection of the walnut gene pool of the Nikitsky Botanical Gardens and analyze genetic relationships based on microsatellite genotyping. On the basis of the performed phenotypic assessment, the study sample, which included 31 varieties, was divided into several groups according to the main phenotypic traits, such as frost and drought resistance, the start of the growing season, the ripening period, the weight and type of flowering, the weight of the fruit, and the thickness of the endocarp. Varieties with economically valuable traits that can be recommended as promising as initial parental forms in breeding work for resistance to abiotic stress factors have been identified, as well as varieties with increased productivity and large fruit sizes. Based on the analysis of eight SSR markers (WGA001, WGA376, WGA069, WGA276, WGA009, WGA202, WGA089 and WGA054), an analysis of the level of genetic diversity was performed and genetic relationships were established in the studied sample of varieties. Six (for WGA089) to eleven (for WGA276) alleles per locus have been identified. A total of 70 alleles were identified for the eight DNA markers used, with an average value of 8.75. Analysis of SSR genotyping data using Bayesian analysis established the presence of two main groups of genotypes. Taking into account the fact that all the studied varieties are selections from local seed populations in different regions of the Crimean Peninsula, the revealed level of polymorphism may indirectly reflect the level of genetic diversity of the local walnut populations. Furthermore, the presence of two genetically distant groups indicates the presence of two independently formed pools of the autochthonous gene pool of the species Juglans regia L. on the Crimean Peninsula

## Abstract (ru)

Орех грецкий – важная садовая культура, которая по объему производства занимает второе место среди всех орехоплодных. Несмотря на значительную потребность на внутреннем рынке, промышленное производство плодов ореха грецкого в России в настоящее время развито недостаточно. При этом существует необходимость обновления сортимента новыми высокопродуктивными сортами, адаптированными к местным агроклиматическим условиям и обладающими высоким качеством плодов, конкурентоспособными на мировом уровне. Важным вопросом для успешной реализации селекционных программ является комплексное изучение генофонда. В связи с этим в рамках исследования была поставлена задача оценки по комплексу признаков перспективных сортов из коллекции генофонда ореха грецкого Никитского ботанического сада и анализа гене- тических взаимосвязей на основе микросателлитного генотипирования. По результатам выполненной феноти- пической оценки изучаемая выборка, включающая 31 сорт, была разделена на несколько групп по основным хозяйственно-биологическим характеристикам, таким как морозо- и засухоустойчивость, срок начала вегета- ции, сроки созревания, тип цветения, масса плода, толщина эндокарпа. Выделены сорта с хозяйственно ценны- ми признаками, которые можно рекомендовать как перспективные в качестве исходных родительских форм в селекционной работе на устойчивость к абиотическим стресс-факторам, а также сорта с повышенной урожайно- стью и обладающие крупным размером плодов. На основании анализа восьми SSR-маркеров (WGA001, WGA376, WGA069, WGA276, WGA009, WGA202, WGA089 и WGA054) оценен уровень генетического разнообразия и опре- делены генетические взаимосвязи в изученной выборке сортов. Выявлено наличие от шести (WGA089) до один- надцати (WGA276) аллелей на локус. Суммарно по восьми использованным ДНК-маркерам было идентифициро- вано 70 аллелей при среднем значении 8.75. Анализ данных SSR-генотипирования в программе Structure 2.3.4 установил наличие двух основных групп генотипов. С учетом того, что все изученные сорта представляют собой отборы из местных семенных популяций в разных районах Крымского полуострова, выявленный уровень по- лиморфизма может опосредованно отражать уровень генетического разнообразия местного генофонда ореха грецкого. При этом наличие двух генетически обособленных групп, вероятно, свидетельствует о существовании двух независимо сформировавшихся пулов автохтонного генофонда вида Juglans regia L. на Крымском полу- острове

## Introduction

Walnut is one of the most important nut crops, which is second only to almonds in terms of production. The world leaders in the production of walnuts are China, Iran, the USA and Turkey (Vahdati et al., 2019). Commercial production of walnut fruits in Russia is currently not developed, however, there are positive trends in the establishment of industrial orchards. At the same time, there is a need to update the assortment with new highly productive varieties adapted to local agro-climatic conditions and having high quality fruits

Obviously, a comprehensive study of the walnut gene pool is an important issue for increasing the efficiency of using genetic resources in solving breeding problems to create new generation varieties, as well as for preserving and replenishing collections. At the same time, the assessment of the level of genetic diversity, elucidation of the degree of genetic similarity, as well as DNA certification of collection samples occupy an important place. One of the most popular methods for assessing the genetic diversity of the walnut is the analysis of microsatellite loci of the genome (Vahdati et al., 2019). SSR markers currently widely used for the analysis of walnut polymorphism were developed both directly for this species (Dangl et al., 2005; Topcu et al., 2015; Ikhsana et al., 2016) and for the species Juglans nigra L. (Woeste et al., 2002). Subsequently, these markers were effectively used to study the interspecific diversity of J. regia due to the high level of cross-reproducibility within the Juglans genus. At the same time, it is worth highlighting the SSR markers marked as “WGA”, which are the most commonly used (Bernard et al., 2018b). With the use of SSR markers for walnut, a significant amount of research has been carried out aimed at analyzing the genetic structure of gene pool collections, including varieties, promising breeding forms, as well as selections from local populations of interest for breeding.

The most large-scale studies of the genetic diversity of collections of genetic resources include the work performed by a team of authors from the INRA Research Center, in which, using 13 SSR markers, genotyping of 217 accessions of walnut and 36 accessions of other species of the genus Juglans from the INRA collection was carried out. Based on the SSR genotyping data, the presence of the main two groups of the greatest genetic similarity was established, which for the most part corresponded to the ecological and geographical origin of the varieties. The data obtained made it possible to form a core collection of fifty samples, reflecting the genetic polymorphism of the entire sample (Bernard et al., 2018a). It is noteworthy that a comparative analysis of the effectiveness of using 13 SSR markers in the above work (Bernard et al., 2018a) and 364,275 SNP markers – data obtained using the Axiom™ J. regia 700K SNP genotyping array SNP chip, showed a close level of information content of the two approaches used in assessing the genetic structure of collections (Bernard et al., 2018a, 2020a). A comparable study of a sample of 189 varieties and breeding forms, representative of gene collections from 25 regions in 14 countries of the world, made it possible to establish the presence of two main groups, including accessions from: (1) Europe and North Africa and (2) Greece and the Middle East (Ebrahimi et al., 2016). Along with such large-scale studies, a wide range of studies was performed using microsatellites on gene pool collections, as well as local populations in different regions of the world: Europe (Pollegioni et al., 2011; Ebrahimi et al., 2017b; Vischi et al., 2017; Cseke et al., 2022), East Asia (Gunn et al., 2010; Wang et al., 2015; Zhou et al., 2017), Central and South Asia (Pollegioni et al., 2014; Roor et al., 2017; Shah et al., 2018; Gaisberger et al., 2020; Magige et al., 2022), the Middle East region (Ebrahimi et al., 2011; Shamlu et al., 2018; Orhan et al., 2020; Davoodi et al., 2021; Guney et al., 2021), North America (Dangl et al., 2005; Aradhya et al., 2010; Ebrahimi et al., 2017a)

A number of studies are known in which, along with molecular genetic analysis of polymorphism based on SSR markers, a comprehensive assessment of the phenotypic variability of samples was performed (Ebrahimi et al., 2011), or an assessment of individual groups of traits, such as fruit characteristics (Chen et al., 2014). This made it possible both to compare the efficiency of using different approaches to determine the groups of the greatest genetic similarity (Pop et al., 2013), and to identify selectively valuable accessions at the first stage and subsequently to evaluate the heterogeneity of the selected groups of accessions based on microsatellite analysis data (Karimi et al., 2014; Davoodi et al., 2021).

Despite the ongoing breeding work on walnuts in the south of Russia (Lugovskoi, Murzinova, 2010; Khokhlov, Baskakova, 2015; Suprun et al., 2016; Lugovskoy, Balapanov, 2018) and the availability of research results on the study of collections of varieties using molecular genetic methods (Balapanov et al., 2019), one should still note the limitations of studies aimed at analyzing the level of genetic diversity and identifying the genetic structure of the gene pool in the South of Russia, including the Crimea and the North Caucasus. The Nikitsky Botanical Gardens (NBG-NSC) is one of the leading scientific organizations in the Russian Federation that performs breeding work on walnuts. The collection of genetic resources of the Nikitsky botanical walnut is represented by 76 accessions. The basis is made up of varieties of selection NBG-NSC (86 %). Among the introduced genotypes, 10 % of the total collection falls on varieties from Moldova and 3 % each on accessions from Ukraine, Europe, the USA, and Tajikistan (Khokhlov, Baskakova, 2015). It is obvious that a comprehensive phenotypic assessment, the identification of groups of the most valuable genotypes, characterized by the presence of several breeding-valuable traits at the same time, as well as the analysis of genetic relationships of valuable varieties and forms, will improve the efficiency of the breeding use of the gene pool in order to create new adaptive varieties with increased productivity potential and high fruit quality.

In the presented work, we set the task of assessing perspective varieties of walnut from the collection of the gene pool of the NBG-NSC according to economically valuable traits, identifying groups of varieties with a complex of important characteristics and analyzing their genetic relationships using microsatellite DNA markers.

## Materials and methods

Phenotypic assessment was carried out in the collection plantations of the laboratory of steppe horticulture (LSH) of the NBG-NSC in 2014–2022. 31 samples of walnut selection from the Nikitsky Botanical Gardens were chosen as the object of observation (Khokhlov, 2012). The LSH territory is located 25 km north of Simferopol, in the village of Novy Sad (45°08′50ʺ N, 33°59′55ʺ E), Republic of Crimea, Russia. In the system of agro-climatic zoning of the peninsula, it belongs to the central plain-steppe region, characterized by an arid climate with a moderately hot growing season and mild, unstable winters (Antyufeev et al., 2002). Also, in the genotyping work, the Chandler variety of the USA selection was used. The relief of the area on which the collection garden is located is flat and slightly wavy; the soil of the plot is southern carbonate low-humus heavy loamy chernozem on red-brown Pliocene clays. The average annual air temperature is +10.5 °C, the average January does not exceed –1.0 °C, and the average July, +21.9 °C. Walnut plants are planted according to the scheme 12×12 m, peach is used as a compactor. The aisles are kept under black fallow. The age of the trees is 30 years.

Determination of the degree of frost resistance of varieties was carried out according to the method developed in the Nikitsky Gardens (Rihter, Yadrov, 1981) and the method of Lapin and Ryabova (1982). The assessment of drought resistance of walnut plants was carried out in accordance with methodological recommendations (Eremeev, Lishchuk, 1974; Kushnirenko et al., 1975; Il’nitskiy, 2005).

A modified CTAB method was used for DNA extraction (Rogers, Bendich, 1985). Genotyping of walnut varieties was carried out using 8 SSR markers: WGA001, WGA376, WGA069, WGA276, WGA009, WGA202, WGA089, WGA054 (Woeste et al., 2002; Dangl et al., 2005). PCR was carried out under the following conditions: the concentration of PCR reagents of the mixture: Buffer 1X, dNTP – 0.24 mM, Taq 1U, SSR primers (forward and reverse) – 0.16 μM each, DNA – 40–50 ng. The following PCR parameters were used: 3 min initial denaturation at 94 °C; the next 35 cycles: 20 sec denaturation at 94 °C, 30 sec primer annealing at 58 °C, 40 sec elongation at 72 °C; final elongation for 10 min at 72 °C. The size of the reaction products was analyzed on a Nanofor 05 automatic genetic analyzer

The data were processed using the GeneMarker V3.0.1 program. The following genetic parameters were calculated in the Microsoft Exel GenAlEx 6.503 macro: Na – number of alleles, Na (average) – average number of alleles, Ne – effective number of alleles, I – Shannon diversity index, Ho – observed heterozygosity, He – expected heterozygosity, F – fixation index (Peakall, Smouse, 2012). The PCoA plot with the genetic similarity coefficient Dice was built using the Past 2.17 program (Hammer et al., 2001) based on a binary matrix. Cluster analysis was carried out using the Structure 2.3.4 program. The optimal value of clusters for analysis was established in the Structure Harvester online program (Evanno et al., 2005)

## Research results

Phenotypic evaluation

Based on the comprehensive phenotypic assessment, the studied varieties were combined into several groups according to the main economic and biological characteristics.

By the degree of frost resistance: varieties with high frost resistance, in which 60–100 % of generative and vegetative buds were preserved without damage – ‘Arkad’, ‘Burlyuk’, ‘Orionid’, ‘Skaberi’, ‘Yuzhnoberezhniy’, ‘Pozdnotsvetushchiy’; moderate frost resistance (from 40 to 60 %) – ‘Bospor’, ‘Al’minskiy’, ‘Konkursniy’, ‘Pamyati Pasenkova’, ‘Dolinniy’, ‘Zolotopolyanskiy’, ‘Krymskiy Skoroplodniy’, ‘Zhemchuzhniy’, ‘Sokoliniy’, ‘Novikov’, ‘Bulganak’, ‘Gurzufskiy’, ‘Sladkoyaderniy’, ‘Pioner Kryma’, ‘Bel’bekskiy Ranniy’, ‘Partizanskiy’, ‘Dzerzhinskiy’, ‘Bel’bekskiy’, ‘Komsomolets’, ‘Bomba Chkalovskaya’, ‘Kollektivniy’; low frost resistance (less than 40 %) – ‘Bubenchik’, ‘Kacha’, ‘Malosadoviy’, ‘Podlesniy’.

By the degree of drought resistance: with high stability – Arkad’, ‘Burlyuk, ‘Orionid’, ‘Bel’bekskiy Ranniy’, ‘Zhemchuzhniy’; with stability above average – ‘Al’minskiy’, ‘Bospor’, ‘Konkursniy’, ‘Pamyati Pasenkova’, ‘Zolotopolyanskiy’, ‘Krymskiy Skoroplodniy’, ‘Pozdnotsvetushchiy’, ‘Sokoliniy’, ‘Yuzhnoberezhniy’, ‘Novikov’, ‘Bulganak’, ‘Gurzufskiy’, ‘Sladkoyaderniy’, ‘Dolinniy’, ‘Pioner Kryma’, ‘Kollektivniy’, ‘Partizanskiy’, ‘Dzerzhinskiy’, ‘Bel’bekskiy’, ‘Komsomolets’, ‘Malosadoviy’, ‘Podlesniy’, ‘Skaberi’, ‘Bomba Chkalovskaya’; with stability below average – ‘Bubenchik’, ‘Kacha’.

By maturity: early – Arkad’, ‘Bulganak’, ‘Dolinniy’, ‘Komsomolets’, ‘Krymskiy Skoroplodniy’, ‘Zhemchuzhniy’, ‘Orionid’, ‘Yuzhnoberezhniy’; middle – ‘Al’minskiy’, ‘Novikov’, ‘Gurzufskiy’, ‘Sladkoyaderniy’, ‘Zolotopolyanskiy’, ‘Pamyati Pasenkova’, ‘Burlyuk’, ‘Pozdnotsvetushchiy’, ‘Sokoliniy’, ‘Dzerzhinskiy’, ‘Bospor’, ‘Bubenchik’, ‘Kollektivniy’, ‘Kacha’, ‘Partizanskiy’, ‘Podlesniy’, ‘Pioner Kryma’, ‘Bel’bekskiy Ranniy’, ‘Bomba Chkalovskaya’, ‘Skaberi’, ‘Kollektivniy’; late – Konkursniy’, ‘Malosadoviy’.

By type of flowering: protogeny (male inflorescences bloom first) – ‘Al’minskiy’, ‘Novikov’, ‘Bulganak’, ‘Gurzufskiy’, ‘Sladkoyaderniy’, ‘Dolinniy’, ‘Pioner Kryma’, ‘Bel’bekskiy Ranniy’, ‘Bomba Chkalovskaya’, ‘Skaberi’; protandria (female flowers bloom first) – ‘Bubenchik’, ‘Kollektivniy’, ‘Kacha’, ‘Partizanskiy’, ‘Konkursniy’, ‘Dzerzhinskiy’, ‘Bel’bekskiy’, ‘Komsomolets’, ‘Malosadoviy’, ‘Podlesniy’; homogamy (simultaneous flowering of male inflorescences and female flowers) – ‘Zolotopolyanskiy’, ‘Arkad’, ‘Krymskiy Skoroplodniy’, ‘Zhemchuzhniy’, ‘Pamyati Pasenkova’, ‘Burlyuk’, ‘Pozdnotsvetushchiy’, ‘Sokoliniy’, ‘Yuzhnoberezhniy’, ‘Bospor’, ‘Orionid’.

By fruit weight: large-fruited (more than 12 g, belong to the variety J. regia L. var. macrocarpa DC. or J. regia f. maxima) – ‘Bomba Chkalovskaya’, ‘Bulganak’, ‘Dolinniy’, ‘Pioner Kryma’, ‘Bel’bekskiy Ranniy’, ‘Skaberi’, ‘Komsomolets’, ‘Malosadoviy’, ‘Podlesniy’, ‘Arkad’, ‘Krymskiy Skoroplodniy’, ‘Burlyuk’, ‘Pozdnotsvetushchiy’, ‘Sokoliniy’, ‘Bospor’, ‘Orionid’; medium-fruited (from 6 to 12 g) – ‘Al’minskiy’, ‘Zolotopolyanskiy’, ‘Pamyati Pasenkova’, ‘Yuzhnoberezhniy’, ‘Kollektivniy’, ‘Kacha’, ‘Novikov’, ‘Partizanskiy’, ‘Konkursniy’, ‘Dzerzhinskiy’, ‘Bel’bekskiy’, ‘Gurzufskiy’, ‘Sladkoyaderniy’, ‘Zhemchuzhniy’; small-fruited (less than 6 g) – ‘Bubenchik’. In all varieties, with the exception of ‘Bomba Chkalovskaya’, the shape of the fruit is oval-round or ovoid.

According to the thickness of the endocarp: thinshelled (from 1.0 to 1.5 mm, belong to the variety J. regia L. var. tenera DC.) – ‘Zolotopolyanskiy’, ‘Yuzhnoberezhniy’; standard shell (from 1.5 to 2 mm, J. regia f. semidura DC.) – ‘Bomba Chkalovskaya’, ‘Bulganak’, ‘Dolinniy’, ‘Pioner Kryma’, ‘Bel’bekskiy Ranniy’, ‘Skaberi’, ‘Partizanskiy’, ‘Komsomolets’, ‘Malosadoviy’, ‘Podlesniy’, ‘Arkad’, ‘Krymskiy Skoroplodniy’, ‘Burlyuk’, ‘Pozdnotsvetushchiy’, ‘Sokoliniy’, ‘Bospor’, ‘Orionid’, ‘Bubenchik’, ‘Kollektivniy’, ‘Al’minskiy’, ‘Novikov’, ‘Gurzufskiy’, ‘Sladkoyaderniy’, ‘Pamyati Pasenkova’, ‘Zhemchuzhniy’; hard-shelled (more than 2.0 mm, belong to the variety J. regia L. var. dura DC.) – ‘Kacha’, ‘Partizanskiy’, ‘Konkursniy’, ‘Dzerzhinskiy’.

By the beginning of the growing season: early – ‘Dolinniy’, ‘Komsomolets’, ‘Arkad’, ‘Zhemchuzhniy’, ‘Bel’bekskiy Ranniy’; medium – ‘Al’minskiy’, ‘Novikov’, ‘Bulganak’, ‘Gurzufskiy’, ‘Sladkoyaderniy’, ‘Pioner Kryma’, ‘Bomba Chkalovskaya’, ‘Skaberi’, ‘Bubenchik’, ‘Kollektivniy’, ‘Kacha, ‘Partizanskiy’, ‘Dzerzhinskiy’, ‘Bel’bekskiy’, ‘Malosadoviy’, ‘Podlesniy’, ‘Zolotopolyanskiy’, ‘Pamyati Pasenkova’, ‘Burlyuk’, ‘Sokoliniy’, ‘Bospor’, ‘Orionid’, ‘Yuzhnoberezhniy’, ‘Krymskiy Skoroplodniy’; late – ‘Pozdnotsvetushchiy’, ‘Konkursniy’.

The results of a long-term study of the walnut gene pool make it possible to identify varieties with economically valuable traits that can be recommended as initial parental forms in breeding work: for tolerance to abiotic stresses during winter-spring period – ‘Arkad’, ‘Burlyuk’, ‘Orionid’, ‘Skaberi’, ‘Yuzhnoberezhniy’, ‘Pozdnotsvetushchiy’; for increased and high drought resistance – ‘Arkad’, ‘Burlyuk’, ‘Orionid’, ‘Bel’bekskiy Ranniy’, ‘Zhemchuzhniy’. For introduction into production, varieties with complex resistance to adverse climatic conditions are recommended – ‘Burlyuk’, ‘Bospor’, ‘Arkad’, ‘Al’minskiy’, ‘Pamyati Pasenkova’, ‘Orionid’, ‘Konkursniy’, as well as those characterized by high yield and large fruits – ‘Bulganak’, ‘Dolinniy’, ‘Pioner Kryma’, ‘Bel’bekskiy Ranniy’, ‘Skaberi’, ‘Partizanskiy’, ‘Komsomolets’, ‘Malosadoviy’, ‘Podlesniy’, ‘Arkad’, ‘Krymskiy Skoroplodniy’, ‘Burlyuk’, ‘Pozdnotsvetushchiy’, ‘Sokoliniy’, ‘Bospor’, ‘Orionid’.

Analysis of genetic diversity

In order to establish genetic relationships within the studied sample of varieties, analyze the level of genetic diversity and identify the groups of the closest genetic relationship, an analysis of the polymorphism of microsatellite loci was performed.

As a result of microsatellite genotyping, DNA profiles specific for all studied varieties were obtained. Six (WGA089) to eleven (WGA276) alleles per locus have been identified. A total of 70 alleles were identified for the eight DNA markers used, with an average value of 8.75. Analysis of the level of genetic polymorphism included the indicators presented in Table 1.

**Table 1.**
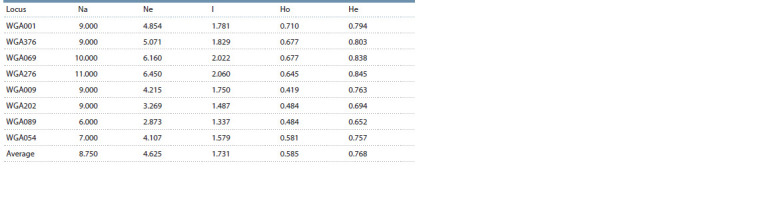
Level of polymorphism of SSR markers Note. Na is the number of identified alleles; Ne is the number of effective alleles; I – diversity index; Ho – observed heterozygosity; He – expected heterozygosity

The value of the Ne index varied from 2.873 (WGA089) to 6.450 (WGA276). At the same time, in the group of markers with the same number of identified alleles (9 alleles per locus): WGA001, WGA376, WGA009, and WGA202, this indicator varied from 3.269 (WGA202) to 5.071 (WGA376), which may be due to variation in allele frequencies. The lowest (1.337) and highest (2.060) values of the diversity index I were found in the least polymorphic marker (WGA089) and the most polymorphic marker (WGA276), respectively. At the same time, the highest value of the observed heterozygosity was found for the WGA001 marker, and the expected heterozygosity, for the most polymorphic WGA276 marker.

**Fig. 1.**
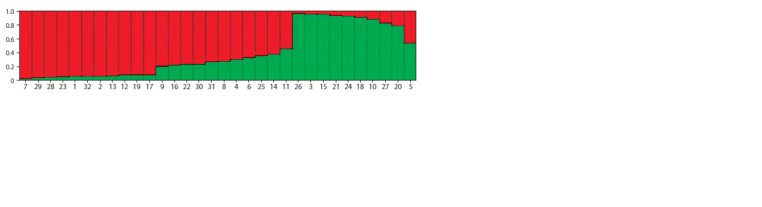
Graph constructed in the Structure program based on genotyping data of eight SSR markers of 32 walnut varieties. Varieties: 1 – ‘Al’minskiy’, 2 – ‘Novikov’, 3 – ‘Bulganak’, 4 – ‘Bubenchik’, 5 – ‘Arkad’, 6 – ‘Gurzufskiy’, 7 – ‘Sladkoyaderniy’, 8 – ‘Zolotopolyanskiy’, 9 – ‘Krymskiy Skoroplodniy’, 10 – ‘Dolinniy’, 11 – ‘Kollektivniy’, 12 – ‘Zhemchuzhniy’, 13 – ‘Konkursniy’, 14 – ‘Kacha’, 15 – ‘Partizanskiy’, 16 – ‘Pamyati Pasenkova’, 17 – ‘Pozdnotsvetushchiy’, 18 – ‘Pioner Kryma’, 19 – ‘Dzerzhinskiy’, 20 – ‘Sokoliniy’, 21 – ‘Bel’bekskiy’, 22 – ‘Bospor’, 23 – ‘Komsomolets’, 24 – ‘Bel’bekskiy Ranniy’, 25 – ‘Bomba Chkalovskaya’, 26 – ‘Malosadoviy’, 27 – ‘Podlesniy’, 28 – ‘Skaberi’, 29 – ‘Yuzhnoberezniy’, 30 – ‘Burlyuk’, 31 – ‘Orionid’, 32 – ‘Chandler’.

Based on the genotyping data of 32 walnut varieties for eight SSR markers, an analysis was carried out using the Structure 2.3.4 program. The range of analyzed clusters was from 2 to 7. Based on the results of the analysis in the Structure Harvester online program, the optimal cluster value was calculated equal to 2. The results obtained with a value of K = 2 are shown in Fig. 1.

According to the predominance of the first or second clusters, the studied Crimean varieties can be conditionally divided into two groups. The first group (the predominance of cluster 1): ‘Bulganak’, ‘Arkad’, ‘Dolinniy’, ‘Partizanskiy’, ‘Pioner Kryma’, ‘Sokoliniy’, ‘Bel’bekskiy’, ‘Bel’bekskiy Ranniy’, ‘Malosadoviy’, ‘Podlesniy’. The second group (the predominance of cluster 2): ‘Al’minskiy’, ‘Novikov’, ‘Bubenchik’, ‘Gurzufskiy’, ‘Sladkoyaderniy’, ‘Zolotopolyanskiy’, ‘Krymskiy Skoroplodniy’, ‘Kollektivniy’, ‘Zhemchuzhniy’, ‘Konkursniy’, ‘Kacha’, ‘Pamyati Pasenkova’, ‘Pozdnotsvetushchiy’, ‘Dzerzhinskiy’, ‘Bospor’, ‘Komsomolets’, ‘Bomba Chkalovskaya’, ‘Skaberi’, ‘Yuzhnoberezniy’, ‘Burlyuk’, ‘Orionid’. Variety ‘Chandler’ was assigned to the second group of varieties. It should be noted that some varieties of the second group have a significant contribution from the first cluster (from 0.185 to 0.481), on the other hand, among the varieties assigned to the first group, two varieties have a significant contribution from the second cluster (0.215 and 0.448).

**Fig. 2.**
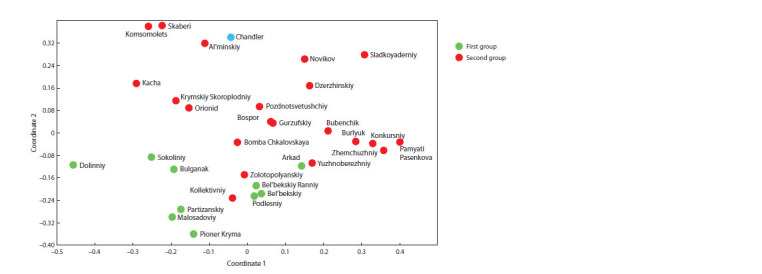
Estimation of genetic relationship by the method of principal coordinates of walnut varieties according to SSR genotyping data.

For a detailed analysis of the genetic relationship of the studied walnut genotypes, an analysis was carried out by the method of principal coordinates (PCoA) in the PAST 2.17 program (Fig. 2).

The distribution of varieties on the PCoA plot largely reflects the grouping of varieties obtained in the Structure program. Varieties of the first group are concentrated at the bottom of the graph. In turn, the varieties of the second group are distributed in the middle and upper parts of the graph. In the arrangement of varieties of the first group, subgroups can be distinguished: (1) varieties ‘Bel’bekskiy Ranniy’, ‘Bel’bekskiy’ and ‘Podlesniy’, (2) ‘Partizanskiy’, ‘Malosadoviy’, ‘Pioner Kryma’, (3) ‘Sokoliniy’, ‘Bulganak’. Two varieties from the first group were not included in one of the subgroups: variety ‘Arkad’ occupies an intermediate position between varieties of the first and second groups, variety ‘Dolinniy’ is equidistant from other varieties included in the first group

Varieties of the second group are distributed on the graph less orderly and do not form clear structures, however, it is worth noting that the varieties ‘Kollektivniy’, ‘Zolotopolyanskiy’, ‘Bomba Chkalovskaya’ and ‘Yuzhnoberezniy’ occupy an intermediate position between the varieties of the first and second groups. Variety ‘Chandler’ on the graph of principal coordinates is spatially close to variety ‘Al’minskiy’.

## Discussion

The SSR markers used in our work were previously widely used to solve various problems in walnut genetics and breeding, including DNA fingerprinting and analysis of the genetic diversity of cultivar collections, breeding-promising forms, and interspecific hybrids (Woeste et al., 2002; Pollegioni et al., 2009; Ebrahimi et al., 2016; Vahdati et al., 2019), study of traitrelated collections (Ebrahimi et al., 2017a), clarification of the issues of gene pool formation within its natural habitats, as well as distribution pathways in the process of domestication (Pollegioni et al., 2014, 2015, 2017).

Comparison of the average values of indicators characterizing polymorphism, identified by the results of our work, and in studies conducted on collections of walnut varieties from other regions of the world, makes it possible to compare the heterogeneity of the studied samples of varieties with that studied in this work. Thus, in a study of a collection of 35 varieties of Chinese breeding using 10 SSR markers, the average Na and Ne values were 9.4 and 4.67, respectively, while the values of expected (He) and observed (Ho) heterozygosity were 0.77 and 0.62, respectively (Chen et al., 2014). In the work of M. Aradhya et al. (Aradhya et al., 2010), when analyzing the genetic polymorphism of a collection of 236 varieties from different walnut growing regions using 15 microsatellite markers, the average number of identified alleles per locus was 11, while the average He and Ho values were 0.699 and 0.536, respectively. It is worth noting that in this study, markers WGA001, WGA202, WGA009, and WGA069 showed higher polymorphism (12, 19, 11, and 13 alleles per locus, respectively), while WGA089 was one of the least polymorphic (8 alleles) (Aradhya et al., 2010). We obtained similar results in terms of the level of allelic polymorphism of markers (see Table 1).

In an analysis of a sample of 189 varieties representing the gene pool of 14 countries, an average of 11.5 alleles per locus was identified, while the average values of observed and expected heterozygosity were 0.62 and 0.73 (Ebrahimi et al., 2016). In this work, the markers WGA001, WGA202, and WGA276, as well as in our study, were included in the group of more polymorphic ones, and the WGA068 marker showed a lower level of polymorphism. In the work of Turkish researchers who performed genotyping of 30 elite breeding forms (candidates for varieties) using 21 SSR markers, an average of 6.15 alleles per locus were identified, while the observed and expected heterozygosity was 0.64 and 0.62 (Bozhuyuk et al., 2020).

Considering the works aimed at studying the genetic diversity of natural populations and promising for breeding forms selected from them, one can also speak of a comparable level of polymorphism. For example, in a study by F. Shamlu et al. (2018), when assessing polymorphism and genetic relationships in a sample of 39 walnut accessions selected in natural populations in northeastern Iran, the average number of identified alleles per locus was 7.9, and the number of effective alleles was 3.91. Expected and observed heterozygosity values were 0.74 and 0.93, respectively. At the same time, the diversity index was lower than the indicator we identified, 1.34 (Shamlu et al., 2018).

In studies devoted to the analysis of the structure of natural populations, elucidation of the ways in which the gene pool spreads, and the formation of its local pools, the indicators of the number of alleles varied. In a study of the genetic diversity of local populations in the Eastern Alps of Italy (Vischi et al., 2017), the average number of alleles per locus was 4.7 in a sample of 13 markers (WGA class) and 2.7 in a sample of seven EST-SSR markers, which is a rather low figure, especially given the sample size of about 200 samples. This can be explained by the possible isolation of the studied population, selected in mountainous areas, as well as in the flat area limited by them (Vischi et al., 2017). In studies of natural walnut populations in Southwestern Tibet, when analyzing a sample of 86 genotypes selected in five geographical locations, the Na indicator was 9.92, but the Ne value was 3.95, which may be due to an uneven distribution of allele frequencies (Wang et al., 2015). In a study aimed at studying the ways of formation and distribution of the walnut gene pool from the centers of its origin in Eurasia, as a result of SSR genotyping of a sample of about 2000 genotypes using 14 SSR markers, an average allele number per locus was found to be 14.21 (Pollegioni et al., 2017).

In general, considering the work on the study of the genetic polymorphism of the walnut gene pool, both cultural forms (varieties, hybrids, selections from local populations) and local populations, including natural ones in regions related to the centers of origin of the species J. regia L., we can make a conclusion about the high level of polymorphism of the sample of varieties studied by us. Given the fact that all the varieties studied are selections from local seed populations in different regions of the Crimean Peninsula, this level of polymorphism may indirectly reflect the level of genetic diversity of the local walnut gene pool. This is confirmed to a certain extent by the results obtained by us in the course of cluster analysis. Based on the data obtained, it can be concluded that the autochthonous gene pool of the walnut probably comes from two hypothetical populations. The division of the studied sample of varieties into two groups based on the results of Bayesian analysis is consistent with the high level of polymorphism, since the presence of two genetically distinct groups may contribute to a higher level of genetic heterogeneity, including allelic polymorphism of DNA markers

In the work, the groups identified during clustering were compared according to a number of population genetic parameters (Table 2).

**Table 2.**
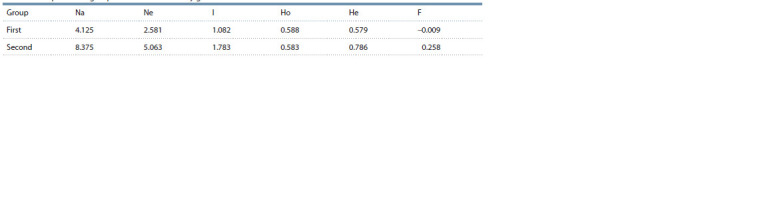
Comparison of groups of walnut varieties by genetic characteristics

The value of the average number of alleles per locus in the second group is two times higher than this indicator in the first group; such indicators of genetic diversity as the effective number of alleles and the Shannon diversity index also reflect a greater allelic polymorphism of microsatellite markers in the second group of varieties. The observed heterozygosity in the groups has a similar value of 0.583 and 0.588, in turn, the expected heterozygosity is higher in the second group. The fixation index has a low positive value in the second group of varieties; in the varieties of the first group, the parameter tends to zero.

At the genetic level, the differences between the groups, in addition to the specific allelic composition of SSR loci, characteristic of each sample of varieties, are also expressed in the degree of allelic diversity. The second group of varieties significantly exceeds the first group of varieties in terms of a number of genetic parameters that reflect the degree of allelic diversity. It can be assumed that the first group of varieties is represented by the most isolated part of the autochthonous gene pool of the Crimean Peninsula. In turn, the genetic diversity of the second group of varieties was influenced by the genetic contribution of the introduced gene plasma brought into the region from outside. An additional confirmation of this assumption is the assignment of the ‘Chandler’ variety, US selection, to the second group. The value of the fixation index in the first group of varieties is typical for populations in a state of panmixia and the absence of genetic barriers that increase the number of observed homozygotes. The data obtained in the Structure 2.3.4 program indicate a genetic relationship between the groups, expressed in the presence of samples with a comparable contribution of two clusters. Since the studied varieties are forms selected in the walnut population that exists within the borders of the Crimean Peninsula, it can be assumed that the high values of the observed heterozygosity reflect the significant size of the walnut gene pool, which contributes to panmixia. In general, the first group of walnut varieties is characterized by high yields and large fruits (more than 12 g).

## Conclusion

Based on a comprehensive phenotypic assessment of samples from the collection of the walnut gene pool of the Nikitsky Botanical Gardens, groups of cultivars with a complex of economically valuable traits were identified. When performing microsatellite genotyping, a high level of genetic diversity and the presence of two genetically distinct groups of varieties were established. One of the groups includes predominantly large-nut cultivars with increased productivity potential, which actualizes the use of them as breeding material, and their genetic remoteness from the rest of the gene pool can increase the effect of heterosis during hybridization.

## Conflict of interest

The authors declare no conflict of interest.
